# Development and Validation of a Thoracic Aortic Calcification-Based Nomogram for Early Myocardial Injury in Breast Cancer Patients

**DOI:** 10.3390/jcdd13090445

**Published:** 2026-09-08

**Authors:** Lingqu Zhou, Liangjiao Wang, Junjie Wang, Zirui Zhou, Xiuquan Zhang, Ziyue Zhong, Qi Guo, Yinyin Zhang

**Affiliations:** 1 Department of Ultrasonography and Electrocardiograms, State Key Laboratory of Oncology in South China, Collaborative Innovation Center for Cancer Medicine, Sun Yat-sen University Cancer Center, Guangzhou 510060, China; zhoulq1@sysucc.org.cn (L.Z.);; 2 Department of Cancer Prevention, Sun Yat-sen University Cancer Center, Guangzhou 510060, China; wangjj278@mail2.sysu.edu.cn; 3 State Key Laboratory of Oncology in South China, Collaborative Innovation Center for Cancer Medicine, Guangdong Key Laboratory of Nasopharyngeal Carcinoma Diagnosis and Therapy, Sun Yat-sen University Cancer Center, Guangzhou 510060, China; 4 Department of Cardiology, Sun Yat-sen Memorial Hospital, Sun Yat-sen University, Guangzhou 510060, China

**Keywords:** cardio-oncology, breast cancer, vascular calcification, myocardial injury, risk prediction

## Abstract

Early identification of patients with breast cancer who are at increased risk of treatment-related myocardial injury may support individualized cardiovascular surveillance. This retrospective study included 551 patients treated at Sun Yat-sen Memorial Hospital between January 2014 and December 2021. All patients had chest computed tomography data and at least three high-sensitivity cardiac troponin T (hs-cTnT) measurements. Early myocardial injury was defined as hs-cTnT > 14 ng/L within 6 months after treatment. Patients were randomly divided into training (*n* = 366) and validation (*n* = 185) cohorts. Candidate predictors were identified using univariable Cox regression and least absolute shrinkage and selection operator regression. A multivariable Cox model was then used to construct the nomogram. Baseline hs-cTnT, high-density lipoprotein cholesterol, thoracic aortic calcification score, and anti-human epidermal growth factor receptor 2 therapy were retained as independent predictors. In the validation cohort, the nomogram showed good discrimination, with an area under the receiver operating characteristic curve of 0.901 and a concordance index of 0.882. Calibration curves indicated agreement between predicted and observed risks. Decision curve analysis suggested clinical utility, while risk stratification identified distinct groups in both cohorts (*p* < 0.001). This thoracic aortic calcification-based nomogram may support the identification of patients at high risk of early myocardial injury after anticancer therapy.

## 1. Introduction

Cardio-oncology focuses on the prevention, detection, and management of cardiovascular complications in patients with cancer [1,2]. Breast cancer is the most common malignancy among women worldwide, and advances in screening and anticancer therapy have substantially improved survival [3,4]. Despite these advances, cardiovascular toxicity remains a major challenge in breast cancer care [5]. Myocardial injury, reflected by elevated cardiac troponin levels during or after treatment, is an early manifestation of cancer therapy-related cardiovascular toxicity (CTR-CVT) [1,6]. High-sensitivity cardiac troponin T (hs-cTnT) is widely available and can detect subclinical myocardial injury before overt cardiac dysfunction. This biomarker may therefore support early risk stratification and individualized surveillance [7,8,9].

Imaging markers of subclinical vascular damage may complement conventional risk factors in individualized cardiovascular risk assessment [10]. Vascular calcification is an imaging marker of subclinical atherosclerosis and cardiovascular vulnerability and has been associated with future cardiovascular events and mortality [11]. In patients with breast cancer, coronary artery calcification (CAC) and thoracic aortic calcification (TAC) can often be assessed on chest computed tomography (CT) performed for pretreatment staging, radiotherapy planning, or other clinical indications [12,13]. These imaging findings may provide additional information on baseline cardiovascular risk before anticancer treatment. However, evidence remains limited regarding the association between baseline vascular calcification and subsequent myocardial injury after breast cancer treatment. Few prediction models have incorporated vascular calcification when assessing myocardial injury risk in this population.

Nomograms are practical prediction tools that integrate multiple clinical variables to estimate individualized risk [14]. They have been widely used in oncology and cardiovascular research to support risk assessment and clinical decision-making [15,16]. Therefore, a nomogram incorporating clinically available variables may help predict the risk of myocardial injury after breast cancer treatment. We aimed to identify predictors of myocardial injury within 6 months after breast cancer treatment and to develop and validate a multivariable Cox regression-based prognostic nomogram. The model was developed in a training cohort and evaluated in a validation cohort for discrimination, calibration, and clinical utility.

## 2. Methods

### 2.1. Data Source and Study Population

We retrospectively screened patients with breast cancer admitted to Sun Yat-sen Memorial Hospital between January 2014 and December 2021. Patients were eligible if they had at least three hs-cTnT measurements and at least 6 months of follow-up. Eligible patients were randomly divided into training and internal validation cohorts at a ratio of 2:1. Patients were further classified according to whether myocardial injury occurred within 6 months after breast cancer treatment. The nomogram was developed using the training cohort and internally validated in the validation cohort.

The exclusion criteria were as follows: (1) unavailable hs-cTnT data at baseline or during follow-up, or baseline hs-cTnT > 14 ng/L; (2) absence of chest CT data; (3) coexisting malignancies or autoimmune diseases requiring immunosuppressive or antitumor-related treatment; (4) pregnancy or lactation; (5) history of myocardial infarction or severe heart failure, or current acute myocardial infarction, myocarditis, severe arrhythmia, severe infection, or renal dysfunction; and (6) age < 18 years or substantial missing data on key clinical variables (Supplementary Figure S1).

### 2.2. Clinical Data Collection

Baseline clinical data were extracted from electronic medical records at initial admission. The collected variables included age, sex, body mass index (BMI), vital signs, admission status, medical history, cancer-related diagnosis, laboratory test results, echocardiographic findings, chest CT findings, and treatment-related information. The primary endpoint was myocardial injury within 6 months after breast cancer treatment. Myocardial injury was defined as an hs-cTnT level > 14 ng/L at any time during follow-up [8,17]. Hs-cTnT was measured using the Elecsys Troponin T hs assay on a cobas e601 analyzer (Roche Diagnostics, Mannheim, Germany). The analytical measurement range was 3–10,000 ng/L, and the 99th-percentile upper reference limit was 14 ng/L. For patients with elevated hs-cTnT during follow-up, medical records around the time of measurement were reviewed, and elevations attributable to acute myocardial infarction, myocarditis, pulmonary embolism, severe infection, acute renal dysfunction, atrial fibrillation, or other clinically significant arrhythmias were not classified as endpoint events.

### 2.3. Calculation of Vascular Calcification Scores

Vascular calcification was assessed using a previously reported noninvasive visual scoring method [18,19,20]. Axial CT images were reviewed, and calcification severity was graded according to the definitions provided in Supplementary Table S1. CAC was assessed separately in the four major coronary artery branches, with each branch assigned a score from 0 to 3. The branch scores were summed to obtain a total CAC score ranging from 0 to 12. TAC was assessed in the ascending aorta, descending aorta, and branches of the aortic arch. The ascending and descending aorta were each scored from 0 to 3, and the aortic arch branches were scored from 0 to 2, yielding a total TAC score ranging from 0 to 8. CAC was categorized as absent, mild, moderate, or severe according to scores of 0, 1–2, 3–5, and ≥6, respectively. TAC was categorized as absent, mild, moderate, or severe according to scores of 0, 1–2, 3–4, and ≥5, respectively. All CT images were evaluated for CAC and TAC by three assessors, including one board-certified radiologist with 10 years of experience and two research physicians with 2 and 3 years of experience in chest CT interpretation. Interobserver agreement for the TAC score was assessed using a two-way random-effects intraclass correlation coefficient (ICC) with absolute agreement, based on the independent scores assigned by the three assessors before consensus resolution. The interobserver agreement for TAC scoring was good, with an ICC of 0.89 (95% CI, 0.87–0.91).

### 2.4. Statistical Analysis

The normality of continuous variables was assessed using the Kolmogorov–Smirnov test. Normally distributed continuous variables were presented as mean ± standard deviation (SD) and compared using Student’s *t* test. Non-normally distributed continuous variables were presented as median (interquartile range) and compared using the Mann–Whitney U test. Categorical variables were presented as numbers and percentages and compared using the chi-square test.

A prognostic model was developed to estimate the risk of myocardial injury within 6 months after breast cancer treatment. Follow-up started on the first day after completion of anticancer treatment and continued until the first documented myocardial injury or censoring at the last event-free assessment or 6 months, whichever came first. In the training cohort, univariable Cox regression analyses were first performed to identify variables associated with 6-month myocardial injury. Associations were reported as hazard ratios (HRs) with corresponding 95% confidence intervals (CIs). Given the limited number of outcome events, univariable *p* < 0.05 screening was initially used as an exploratory dimensionality-reduction step. A total of 12 variables meeting this criterion were entered into least absolute shrinkage and selection operator (LASSO) Cox regression to reduce model complexity and select candidate predictors. Predictors selected by LASSO Cox regression were then entered into a multivariable Cox proportional hazards model to construct the final prediction model. To assess the robustness of this multistep variable-selection procedure and potential selection optimism, a single penalized Cox model was fitted in the entire cohort using all 12 candidate predictors simultaneously, without additional univariable screening or post-LASSO unpenalized refitting. This sensitivity analysis therefore reduced dependence on repeated data-driven selection and unpenalized refitting, although the 12 candidate predictors themselves originated from the initial univariable screening. The penalty parameter was selected using 10-fold cross-validation. The entire modelling procedure, including imputation, cross-validation, penalty selection, and LASSO fitting, was repeated in 1000 bootstrap resamples to assess predictor-selection stability and model optimism. A nomogram was developed based on the final multivariable Cox model to estimate the probability of 6-month myocardial injury.

Model performance was assessed by discrimination, calibration, and clinical utility. Discrimination was evaluated using the 6-month time-dependent area under the receiver operating characteristic curve (AUC) and the concordance index (C-index). Right censoring in the time-dependent AUC analysis was accounted for using inverse probability of censoring weighting (IPCW), with the censoring distribution estimated by the marginal Kaplan–Meier method. Bootstrap resampling with 1000 repetitions was used to derive 95% CIs for the time-dependent AUC and C-index. Calibration curves were used to assess agreement between predicted and observed probabilities of myocardial injury. To further assess model optimism and stability, the final four-variable Cox model was internally validated in the entire cohort using 1000 bootstrap resamples. Model performance was summarized using the optimism-corrected Harrell C-index and calibration slope. Decision curve analysis (DCA) was performed to evaluate clinical utility by quantifying net benefit across a range of threshold probabilities, with a high-risk classification interpreted as prompting intensified cardiovascular surveillance. Patients were stratified into low- and high-risk groups using the median nomogram score derived from the training cohort, with the same cutoff applied to the validation cohort. Kaplan–Meier curves were used to compare myocardial injury-free probability between groups.

All statistical tests were two-sided, and *p* < 0.05 was considered statistically significant. Statistical analyses were performed using R software, version 4.3.5 (R Foundation for Statistical Computing, Vienna, Austria).

## 3. Results

### 3.1. Baseline Characteristics of the Training and Internal Validation Cohorts

Baseline characteristics of patients with breast cancer stratified by the occurrence of myocardial injury are shown in Table 1. A total of 551 patients were included, including 366 in the training cohort and 185 in the validation cohort. Myocardial injury occurred in 42 patients in the training cohort and 19 patients in the validation cohort. The mean follow-up duration was 167.52 ± 48.54 days in the training cohort and 159.73 ± 53.72 days in the validation cohort.

In the training cohort, the mean age was 48.64 ± 9.44 years in patients without myocardial injury and 52.31 ± 14.12 years in those with myocardial injury (*p* = 0.027). Compared with patients without myocardial injury, those with myocardial injury were more often postmenopausal and had higher levels of cytokeratin 19 fragment antigen 21-1 (CYFRA 21-1), and hs-cTnT, but lower levels of high-density lipoprotein cholesterol (HDL-C) and apolipoprotein A-I (ApoA-I) (all *p* < 0.05). Anti-human epidermal growth factor receptor 2 (HER2) therapy was also more frequent in patients with myocardial injury (*p* = 0.001). Baseline CAC and TAC score distributions differed between the two groups in the training cohort (both *p* < 0.001).

In the validation cohort, the mean age was 47.05 ± 9.80 years in patients without myocardial injury and 50.79 ± 12.62 years in those with myocardial injury (*p* = 0.129). Patients with myocardial injury had higher hs-cTnT levels and lower HDL-C levels than those without myocardial injury (both *p* < 0.001). Baseline CAC and TAC score distributions also differed significantly between the two groups (both *p* < 0.001).

### 3.2. Predictor Selection and Multivariable Cox Analysis in the Training Cohort

To develop the prediction model, univariable Cox regression analyses were first performed in the training cohort to identify variables associated with myocardial injury within 6 months after breast cancer treatment. Age, postmenopausal status, use of angiotensin-converting enzyme inhibitors (ACEIs) or angiotensin receptor blockers (ARBs), statin use, anti-HER2 therapy, CA 15-3, HDL-C, ApoA-I, hs-cTnT, follicle-stimulating hormone, CAC score, and TAC score were associated with myocardial injury in the univariable analyses (Supplementary Table S2).

**Figure 1:**
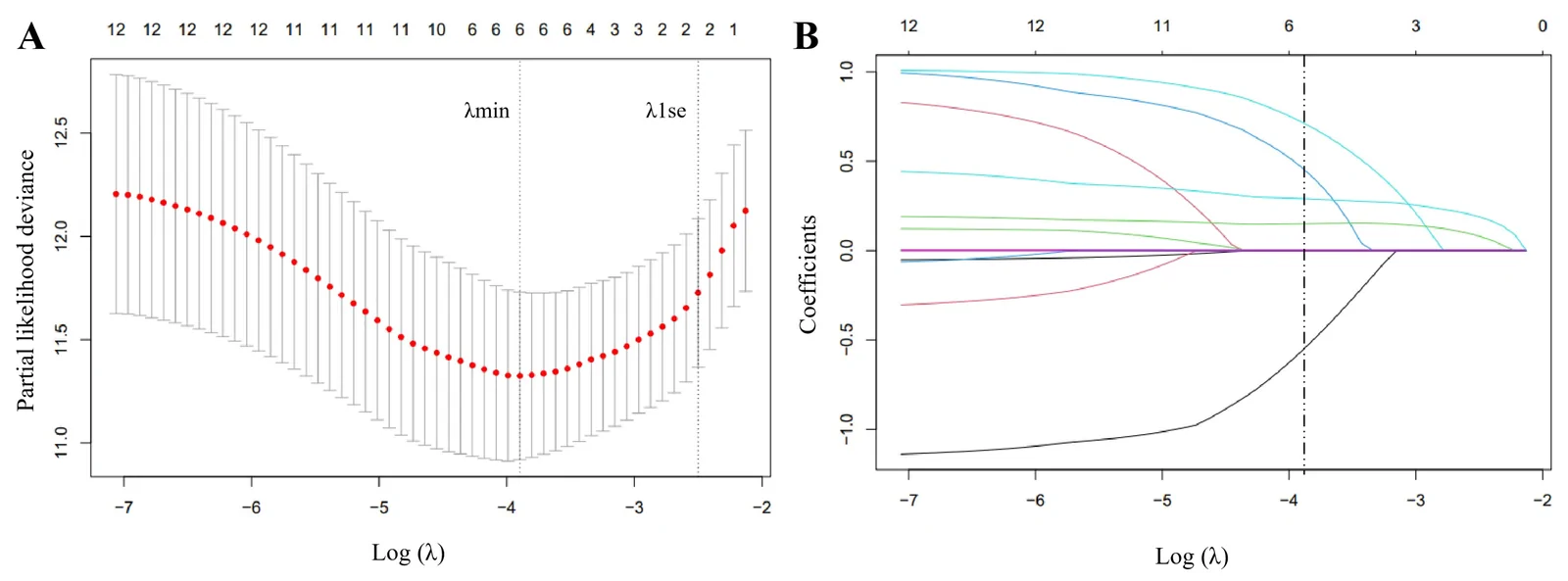
Predictor selection using least absolute shrinkage and selection operator (LASSO) Cox regression in the training cohort. (**A**) Ten-fold cross-validation was used to select the tuning parameter λ. The partial likelihood deviance was plotted against log(λ), and the dotted vertical lines indicate the λ values selected by the minimum criterion and the 1-standard-error criterion. (**B**) Coefficient profiles of candidate predictors across the log(λ) sequence. The vertical dotted line indicates the λ value selected for model development, at which six predictors had nonzero coefficients and were retained for subsequent multivariable Cox proportional hazards regression.

These candidate variables were then entered into a LASSO Cox regression model for further variable selection. Six variables with nonzero coefficients were retained, including hs-cTnT, HDL-C, CA 15-3, CAC score, TAC score, and anti-HER2 therapy (Figure 1).

All selected variables were subsequently included in the multivariable Cox proportional hazards model. In this model, hs-cTnT, HDL-C, TAC score, and anti-HER2 therapy remained independently associated with myocardial injury (Table 2). Higher hs-cTnT levels, higher TAC scores, and anti-HER2 therapy were associated with an increased risk of myocardial injury, whereas higher HDL-C levels were associated with a lower risk. These four predictors were incorporated into the final nomogram. In the supplementary whole-cohort penalized Cox analysis, TAC score, baseline hs-cTnT, HDL-C, and anti-HER2 therapy were retained and showed bootstrap selection frequencies of 99.3%, 96.7%, 88.6%, and 86.7%, respectively. The optimism-corrected C-index was 0.809, and the optimism-corrected calibration slope was 0.946, indicating limited model optimism and generally good stability of the principal predictors.

### 3.3. Development of the Nomogram for 6-Month Myocardial Injury Risk

**Figure 2:**
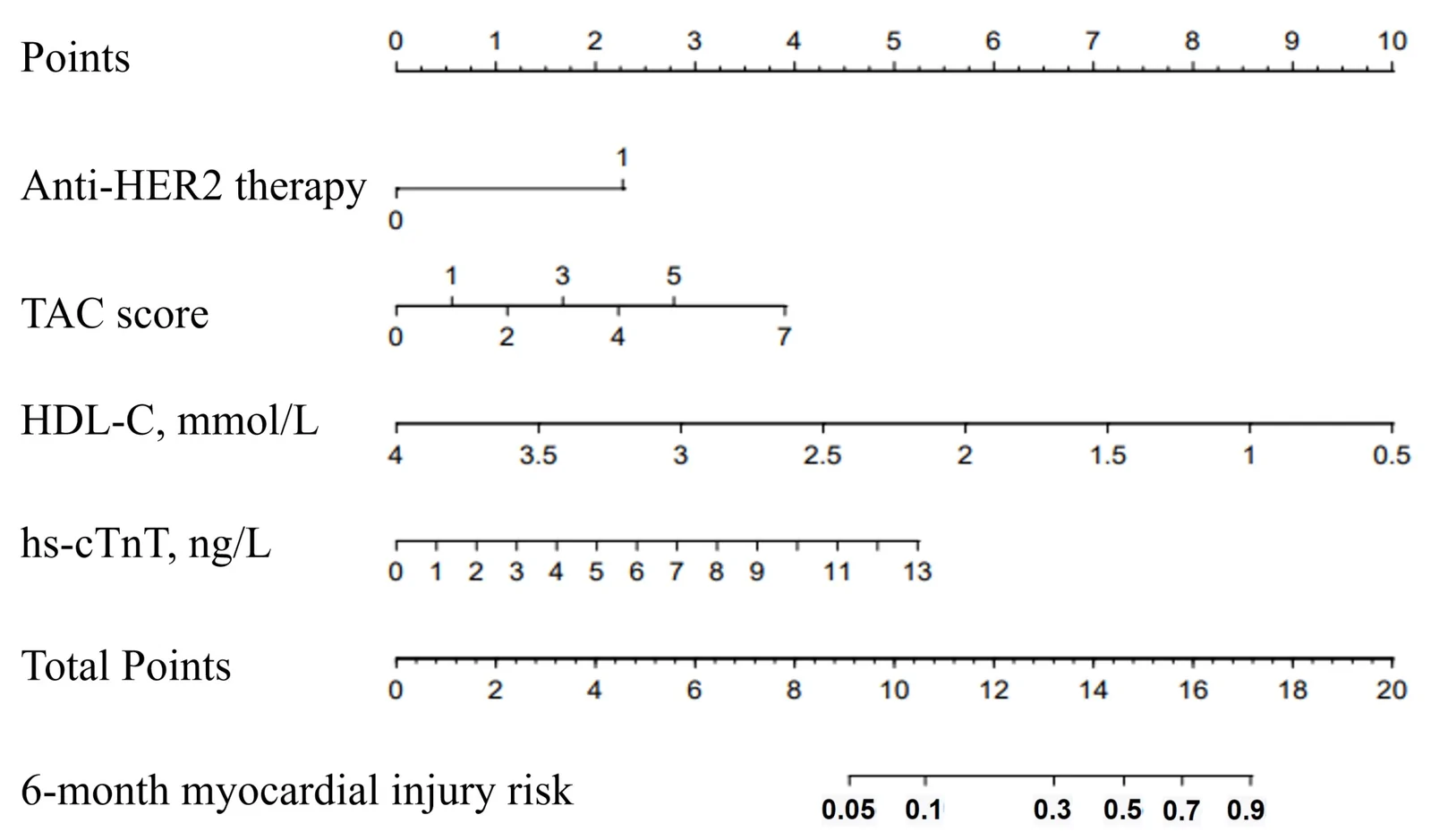
Nomogram for predicting 6-month myocardial injury after breast cancer treatment. The nomogram incorporates anti-human epidermal growth factor receptor 2 (HER2) therapy, thoracic aortic calcification (TAC) score, high-density lipoprotein cholesterol (HDL-C) level, and high-sensitivity cardiac troponin T (hs-cTnT) level. Points assigned to each predictor are summed to generate a total score, which corresponds to the estimated risk of myocardial injury within 6 months after breast cancer treatment.

A prognostic nomogram was constructed based on the four independent predictors identified in the multivariable Cox proportional hazards model: hs-cTnT, HDL-C, TAC score, and anti-HER2 therapy (Figure 2). Each predictor was assigned a corresponding point value according to its contribution to the model. The total points were calculated by summing the points for all predictors and were then mapped to the predicted probability of myocardial injury within 6 months after breast cancer treatment. Higher total points indicated a higher predicted risk of 6-month myocardial injury.

### 3.4. Performance Evaluation of the Prognostic Model

**Figure 3:**
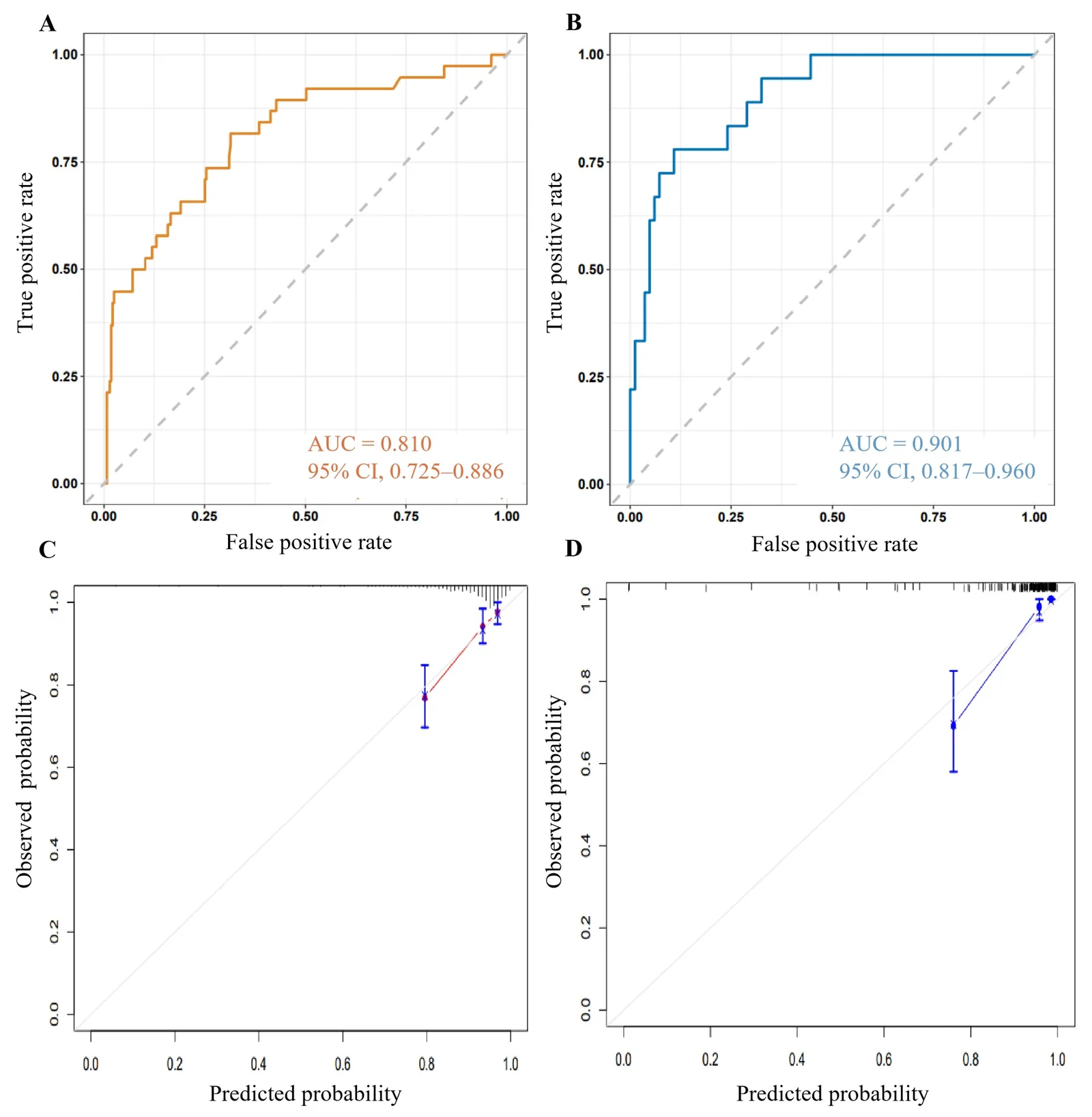
Performance evaluation of the nomogram for predicting 6-month myocardial injury after breast cancer treatment. Receiver operating characteristic curves show the discriminatory performance of the nomogram in the training cohort (**A**) and validation cohort (**B**). Calibration curves show the agreement between predicted and observed probabilities of 6-month myocardial injury in the training cohort (**C**) and validation cohort (**D**). AUC, area under the receiver operating characteristic curve; CI, confidence interval.

The nomogram showed good discrimination for predicting 6-month myocardial injury, with an AUC of 0.810 (95% CI, 0.725–0.886) in the training cohort and 0.901 (95% CI, 0.817–0.960) in the validation cohort (Figure 3A,B). The corresponding C-index values were 0.804 (95% CI, 0.734–0.873) and 0.882 (95% CI, 0.824–0.938), respectively. To assess the incremental predictive value of TAC, we compared Model 1 with Model 2, which additionally included TAC. Model 2 showed modest numerical improvements in discrimination. In the training cohort, addition of TAC increased the C-index from 0.784 to 0.804 (Δ = 0.019) and the AUC from 0.799 to 0.810 (Δ = 0.011). In the internal validation cohort, the corresponding C-index increased from 0.874 to 0.882 (Δ = 0.008), and the AUC increased from 0.870 to 0.901 (Δ = 0.032). The addition of TAC also significantly improved model fit (likelihood-ratio χ^2^ = 6.62, df = 1, *p* = 0.010; Supplementary Table S3). Calibration curves demonstrated good agreement between the predicted and observed probabilities of 6-month myocardial injury in both cohorts (Figure 3C,D). The calibration intercept and slope were 0.043 and 1.000 in the training cohort and 0.247 and 1.314 in the validation cohort, respectively, with corresponding Brier scores of 0.079 and 0.065. Bootstrap internal validation using 1000 resamples yielded an optimism-corrected C-index of 0.824, with an estimated optimism of 0.007. The optimism-corrected calibration slope was 0.908, indicating limited overfitting and satisfactory model stability.

**Figure 4:**
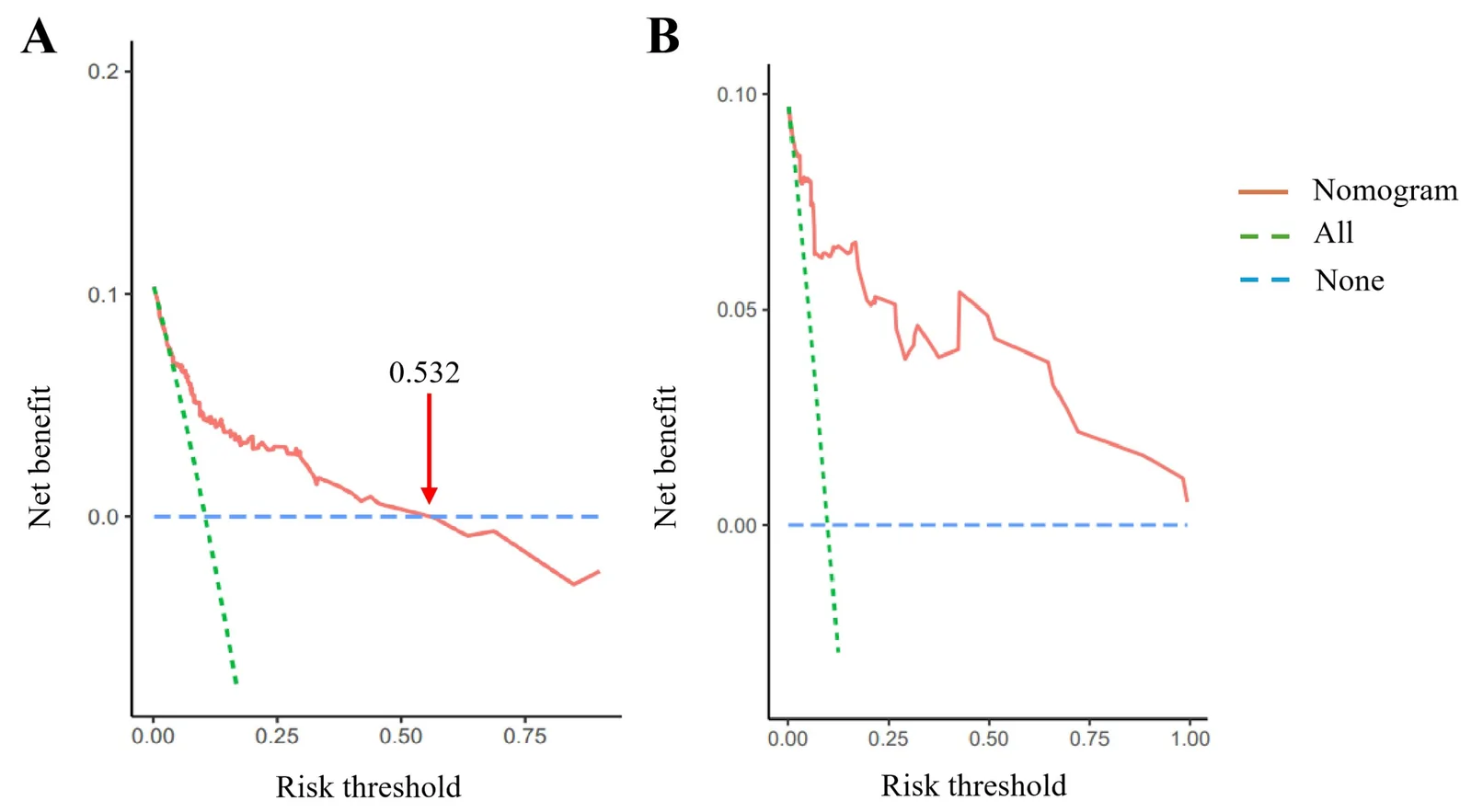
Decision curve analysis of the nomogram in the training and internal validation cohorts. Decision curve analysis was performed to evaluate the clinical utility of the nomogram in the training cohort (**A**) and validation cohort (**B**). The orange solid line represents the nomogram, the green dashed line represents the treat-all strategy, and the blue dashed line represents the treat-none strategy. The nomogram provided a greater net benefit than the treat-all or treat-none strategies across a range of clinically relevant risk thresholds.

**Figure 5:**
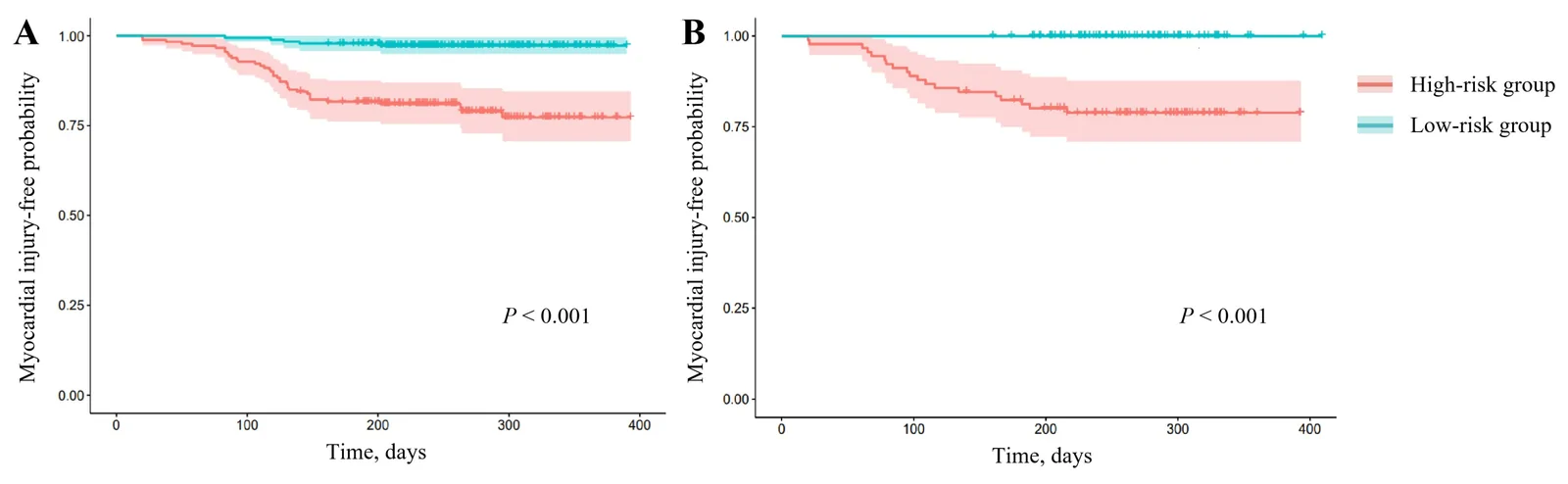
Kaplan–Meier curves of myocardial injury-free probability stratified by nomogram-derived risk groups in the training and internal validation cohorts. Patients were stratified into high- and low-risk groups according to the nomogram-derived total score. Kaplan–Meier curves are shown for the training cohort (**A**) and validation cohort (**B**). Shaded areas represent 95% confidence intervals, and tick marks indicate censored observations. *p* values were calculated using the log-rank test.

Decision curve analysis showed that the nomogram provided a greater net benefit than the treat-all or treat-none strategies across clinically relevant threshold probabilities, particularly when the threshold probability was below 0.532 in the training cohort (Figure 4). In addition, risk stratification based on the nomogram-derived score effectively separated patients into low- and high-risk groups, with significantly lower myocardial injury-free probability in the high-risk group in both cohorts (both *p* < 0.001; Figure 5).

## 4. Discussion

In this retrospective cohort study of patients with breast cancer, we developed and validated a nomogram to estimate the risk of myocardial injury within 6 months after anticancer treatment. The final model incorporated four readily available clinical predictors, including baseline hs-cTnT level, HDL-C level, TAC score, and anti-HER2 therapy. The nomogram demonstrated good discrimination, calibration, and clinical utility in both the training and internal validation cohorts, and effectively stratified patients into distinct risk groups. To our knowledge, this is among the first studies to integrate vascular calcification with clinical and treatment-related factors to predict early myocardial injury after breast cancer treatment.

The 2022 European Society of Cardiology (ESC) cardio-oncology guidelines recommend baseline assessment of CTR-CVT risk using patient characteristics, cancer status, and treatment-related factors [1]. In this context, vascular calcification may provide additional information on pre-existing cardiovascular vulnerability. Previous studies have shown that vascular calcification detected on clinically available CT images, including CAC on radiotherapy-planning CT in patients with breast cancer and CAC or TAC in patients receiving immune checkpoint inhibitor therapy, is associated with subsequent cardiovascular events [12,21]. Consistent with these findings, patients who developed myocardial injury in our study had a higher baseline burden of both CAC and TAC than those without myocardial injury, and both CAC and TAC were associated with 6-month myocardial injury in univariate analyses. After variable selection and multivariable adjustment, TAC remained independently associated with myocardial injury and was included in the final nomogram. CAC primarily reflects localized coronary atherosclerotic plaque burden, whereas TAC may represent a broader phenotype of vascular aging and systemic arterial calcification. This distinction may be particularly relevant in an exclusively female cohort, in which TAC has previously shown cardiovascular prognostic information independent of CAC [22]. Our findings suggest that TAC assessed on available pretreatment chest CT may provide clinically accessible imaging information to complement existing cardiovascular risk assessment for early myocardial injury after breast cancer treatment.

Baseline hs-cTnT was also retained in the final nomogram. In our study, patients who developed myocardial injury had higher baseline hs-cTnT levels, and baseline hs-cTnT remained independently associated with 6-month myocardial injury after multivariable adjustment. This finding is consistent with evidence that elevated troponin levels are associated with subsequent cancer therapy-related cardiac dysfunction, particularly in patients receiving anthracycline-based chemotherapy [23,24]. Its inclusion in the final model supports the use of hs-cTnT as an accessible biomarker for identifying patients at increased risk of early myocardial injury after breast cancer treatment.

HDL-C was inversely associated with 6-month myocardial injury and was retained in the final nomogram. In the Framingham Heart Study, low HDL-C was identified as a strong predictor of subsequent coronary heart disease over long-term follow-up [25]. However, HDL-C concentration does not fully capture HDL particle composition or functional characteristics, which may vary across patients with cancer. In the present study, lower baseline HDL-C was associated with an increased risk of early myocardial injury after breast cancer treatment. These findings suggest that HDL-C may serve as a readily available prognostic marker reflecting baseline cardiometabolic status and help identify patients with increased cardiovascular vulnerability before anticancer treatment. Anti-HER2 therapy was also retained as an independent predictor in the final nomogram. Therapies targeting HER2 are key components of treatment for HER2-positive breast cancer but have been associated with cancer therapy-related cardiac dysfunction, particularly left ventricular systolic dysfunction and heart failure [26,27]. This risk may be further increased by concurrent or sequential exposure to other potentially cardiotoxic therapies, such as anthracycline-based chemotherapy [28]. Experimental evidence indicates that inhibition of HER2 signaling may interfere with ErbB-mediated cardioprotective pathways, thereby reducing myocardial tolerance to cardiotoxic stress [29,30,31]. In the present study, anti-HER2 therapy was associated with an increased risk of 6-month myocardial injury, supporting the inclusion of treatment-related factors in the prediction of biomarker-defined myocardial injury in patients with breast cancer.

This nomogram translates pretreatment cardiovascular assessment into an individualized estimate of early biomarker-defined myocardial injury risk after breast cancer treatment. Current cardio-oncology practice emphasizes baseline risk assessment before potentially cardiotoxic therapy; however, clinical history, biomarkers, imaging findings, and treatment-related factors are not always integrated into a single individualized risk estimate [32]. By integrating routinely available clinical, laboratory, imaging, and treatment-related variables, the nomogram provides a structured framework for identifying patients who may be vulnerable to myocardial injury during the early phase of anticancer treatment. Because early biomarker-defined myocardial injury may precede overt cardiac dysfunction, its prediction may enable risk-adapted surveillance. Patients identified as high risk may require closer hs-cTnT monitoring, timely echocardiographic evaluation, optimization of modifiable cardiovascular risk factors, and early cardio-oncology referral [33]. Conversely, patients at lower predicted risk may be managed with standard guideline-based monitoring, which could support more efficient allocation of cardiovascular surveillance resources. Thus, this nomogram should be viewed as a supportive tool that complements, rather than replaces, clinical judgment and existing cardio-oncology guidelines. Given that the four predictors are routinely available, the nomogram could potentially be converted into an online calculator or integrated into electronic medical records to provide automated individualized risk estimates. Within a cardio-oncology screening pathway, a high predicted risk could prompt closer hs-cTnT monitoring, timely echocardiographic assessment, optimization of cardiovascular risk factors, and cardio-oncology referral. However, external validation, prospective evaluation of clinical impact, and establishment of appropriate risk thresholds are required before routine implementation. Therefore, the nomogram should currently be considered a risk-stratification and decision-support tool rather than a stand-alone trigger for clinical intervention.

Several limitations should be acknowledged. First, this was a single-center retrospective study with a relatively limited sample size, and the validation cohort was generated by randomly splitting patients from the same institutional cohort. Therefore, the model underwent only internal validation, which may limit its generalizability. In addition, eligibility required at least three hs-cTnT measurements, an available chest CT examination, and sufficient follow-up for 6-month outcome ascertainment. These requirements may have preferentially selected patients who underwent more intensive cardiovascular monitoring and had more complete longitudinal clinical data, potentially limiting the representativeness of the study population and introducing selection bias. External validation in independent multicenter cohorts is required before routine clinical application. Second, some potentially important cardiac parameters, including natriuretic peptide biomarkers such as B-type natriuretic peptide or N-terminal pro–B-type natriuretic peptide, as well as global longitudinal strain, were unavailable in a substantial proportion of patients, limiting comprehensive assessment of subclinical cardiac dysfunction [34]. Third, vascular calcification was assessed using a visual scoring method on routine chest CT rather than dedicated electrocardiography-gated calcium scoring, and measurement variability cannot be fully excluded. Fourth, the relatively small number of myocardial injury events (*n* = 61) may have limited model stability and increased the risk of overfitting, despite the use of LASSO-based variable selection and internal validation. Fifth, although hs-cTnT testing frequency did not differ significantly according to anti-HER2 treatment status, the timing of hs-cTnT measurements was not standardized, and testing frequency was not incorporated into a sensitivity analysis; therefore, residual ascertainment bias related to surveillance patterns cannot be excluded. Finally, because treatment information and outcome ascertainment were based on retrospective clinical records, selection bias, information bias, and residual confounding related to treatment regimen, cumulative anthracycline dose, radiotherapy laterality, sequential anthracycline–HER2 exposure, and baseline cardiovascular risk may remain. Future prospective studies are needed to validate this nomogram and to determine whether its use can improve cardiovascular surveillance and outcomes in patients with breast cancer.

## 5. Conclusions

We developed and internally validated a nomogram that estimates the risk of myocardial injury within 6 months after breast cancer treatment. The model included four clinically available predictors: baseline hs-cTnT level, HDL-C level, TAC score, and anti-HER2 therapy. The nomogram showed good predictive performance and may support individualized risk stratification for biomarker-defined myocardial injury and cardiovascular surveillance in patients with breast cancer receiving anticancer treatment.

## Figures and Tables

**Table 1:** Baseline characteristics of patients with breast cancer stratified by myocardial injury status.

	Training Cohort			Validation Cohort		
Variable	No Myocardial Injury (*n* = 324)	Myocardial Injury (*n* = 42)	*p* Value	No Myocardial Injury (*n* = 166)	Myocardial Injury (*n* = 19)	*p* Value
Age, years	48.64 ± 9.44	52.31 ± 14.12	0.027	47.05 ± 9.80	50.79 ± 12.62	0.129
Weight, kg	57.00 (52.00, 64.00)	54.00 (50.00, 60.75)	0.080	55.00 (51.00, 61.00)	56.50 (49.75, 62.00)	0.644
BMI, kg/m^2^	22.67 (21.06, 25.46)	21.97 (20.73, 25.26)	0.426	22.24 (20.14, 24.40)	22.60 (19.69, 26.27)	0.598
Postmenopausal, *n* (%)	130 (40.1)	26 (61.9)	0.012	63 (38.0)	9 (47.4)	0.583
Vital signs at admission						
HR, beats/min	80.00 (76.00, 88.00)	83.50 (73.75, 92.00)	0.384	80.00 (73.25, 89.00)	80.00 (75.50, 94.00)	0.549
SBP, mmHg	121.50 (111.00, 135.25)	120.50 (108.50, 151.50)	0.580	121.50 (112.00, 136.75)	125.00 (113.00, 137.00)	0.578
DBP, mmHg	78.00 (70.75, 83.00)	76.00 (70.00, 83.75)	0.947	77.00 (68.00, 85.00)	78.00 (68.50, 82.50)	0.816
Creatinine, μmol/L	75.61 ± 14.15	84.93 ± 43.38	0.004	81.17 ± 13.09	79.21 ± 13.91	0.540
FBG, mmol/L	5.20 (4.80, 5.70)	5.20 (4.73, 5.60)	0.840	5.30 (4.90, 5.80)	5.50 (5.00, 5.90)	0.610
Calcium, mmol/L	2.32 (2.25, 2.40)	2.31 (2.25, 2.37)	0.460	2.32 (2.25, 2.37)	2.33 (2.20, 2.38)	0.924
Phosphorus, mmol/L	1.20 (1.12, 1.33)	1.20 (1.09, 1.28)	0.652	1.22 (1.13, 1.31)	1.18 (1.14, 1.25)	0.581
HDL-C, mmol/L	1.36 (1.20, 1.65)	1.27 (0.99, 1.41)	<0.001	1.33 (1.21, 1.58)	1.05 (0.97, 1.13)	<0.001
LDL-C, mmol/L	3.18 (2.69, 3.73)	3.22 (2.87, 3.83)	0.430	3.12 (2.62, 3.66)	3.08 (2.50, 3.58)	0.510
ApoA-I, g/L	1.36 (1.18, 1.54)	1.27 (1.17, 1.39)	0.022	1.34 (1.15, 1.55)	1.23 (1.06, 1.38)	0.105
hs-cTnT, ng/L	3.10 (3.00, 3.80)	3.55 (3.00, 7.45)	0.010	3.10 (3.00, 3.60)	3.60 (3.00, 5.45)	<0.001
CA 15-3, U/mL	11.95 (8.30, 19.12)	13.16 (9.93, 22.53)	0.109	12.30 (9.60, 20.00)	15.80 (12.45, 25.50)	0.076
CYFRA 21-1, ng/mL	2.91 (1.93, 3.97)	3.75 (2.13, 5.54)	0.018	2.64 (1.92, 4.04)	3.12 (2.33, 10.85)	0.100
FSH, U/L	10.90 (5.20, 50.60)	17.70 (5.50, 74.30)	0.170	10.20 (4.95, 66.22)	13.20 (3.90, 45.02)	0.602
Anthracycline-based chemotherapy, *n* (%)	252 (78.5)	36 (87.8)	0.236	131 (94.9)	15 (83.3)	0.168
Cyclophosphamide, *n* (%)	265 (82.6)	38 (90.5)	0.281	143 (96.0)	16 (88.9)	0.456
Platinum-based chemotherapy, *n* (%)	41 (13.2)	2 (4.9)	0.201	25 (15.1)	1 (5.3)	0.415
Radiotherapy, *n* (%)	35 (10.8)	3 (7.1)	0.644	8 (4.8)	1 (5.3)	1.000
Anti-HER2 therapy, *n* (%)	85 (26.2)	22 (52.4)	0.001	34 (20.5)	4 (21.1)	1.000
CAC score category, *n* (%)			<0.001			<0.001
None (0)	292 (90.1)	24 (57.1)		147 (88.6)	7 (36.8)	
Mild (1–2)	24 (7.4)	12 (28.6)		13 (7.8)	9 (47.4)	
Moderate (3–5)	8 (2.5)	5 (11.9)		6 (3.6)	2 (10.5)	
Severe (≥6)	0 (0.0)	1 (2.4)		0 (0.0)	1 (5.3)	
TAC score category, *n* (%)			<0.001			<0.001
None (0)	215 (66.4)	18 (42.9)		113 (68.1)	4 (21.1)	
Mild (1–2)	102 (31.5)	13 (31.0)		46 (27.7)	6 (31.6)	
Moderate (3–4)	7 (2.2)	10 (23.8)		7 (4.2)	9 (47.4)	
Severe (≥5)	0 (0.0)	1 (2.4)		0 (0.0)	0 (0.0)	

Data are presented as mean ± SD, median (IQR), or *n* (%), as appropriate. For variables with missing data, descriptive statistics and percentages were calculated based on available observations, with variable-specific nonmissing observations used as the denominator. Abbreviations: ApoA-I, apolipoprotein A-I; BMI, body mass index; CAC, coronary artery calcification; CA 15-3, cancer antigen 15-3; CYFRA 21-1, cytokeratin 19 fragment antigen 21-1; DBP, diastolic blood pressure; FBG, fasting blood glucose; FSH, follicle-stimulating hormone; HDL-C, high-density lipoprotein cholesterol; HER2, human epidermal growth factor receptor 2; HR, heart rate; hs-cTnT, high-sensitivity cardiac troponin T; IQR, interquartile range; LDL-C, low-density lipoprotein cholesterol; SBP, systolic blood pressure; TAC, thoracic aortic calcification.

**Table 2:** Multivariable Cox proportional hazards analysis of predictors of 6-month myocardial injury in the training cohort.

Variable	HR	95% CI	*p* Value
Anti-HER2	2.93	1.58–5.43	0.004
CA 15-3	1.01	1.00–1.02	0.187
HDL-C	0.26	0.09–0.76	0.014
hs-cTnT	1.28	1.12–1.46	<0.001
CAC score	1.12	0.78–1.60	0.534
TAC score	1.30	1.04–1.62	0.019

Abbreviations: CAC, coronary artery calcification; CA 15-3, cancer antigen 15-3; CI, confidence interval; HDL-C, high-density lipoprotein cholesterol; HER2, human epidermal growth factor receptor 2; HR, hazard ratio; hs-cTnT, high-sensitivity cardiac troponin T; TAC, thoracic aortic calcification.

## Data Availability

The data that support the findings of this study are available from the corresponding author, Yinyin Zhang, upon reasonable request.

## Supplementary Materials

The following supporting information can be downloaded at: https://www.mdpi.com/article/10.3390/jcdd13090445/s1, Figure S1: Flowchart of patient selection and study design; Table S1: Visual scoring criteria for vascular calcification on routine diagnostic chest CT; Table S2: Univariable Cox Regression Analysis; Table S3: Incremental predictive value of TAC for 6-month myocardial injury.
