# Supplementary material for: Development and Validation of a Thoracic Aortic Calcification-Based Nomogram for Early Myocardial Injury in Breast Cancer Patients
Source: J Cardiovasc Dev Dis. 2026 Sep 8;13(9):445. doi: 10.3390/jcdd13090445 (PMC13607559; doi:10.3390/jcdd13090445)
Supplement: Supplementary file 1 [file jcdd-13-00445-s001.zip › jcdd-4441262-supplementary.pdf]

Supplementary Table S1. Visual scoring criteria for vascular calcification on routine diagnostic chest CT.

Finding and scoring range	Grade			
	**0**	**1**	**2**	**3**
Aortic wall calcification, scored separately for the ascending and descending aorta (0–3)	Absent	≤3 foci	4–5 foci or 1 calcification extending over 2 slices[a]	>5 foci or 1 calcification extending over ≥3 slices[a]
Supra-aortic artery calcification (0–2)	Absent	Calcification in 1 supra-aortic artery	Calcifications in >1 supra-aortic artery	Not applicate
Coronary artery calcification, scored separately for each major coronary artery branch: LM, LAD, LCX, and RCA (0–3)	Absent	1–2 foci	>2 foci or 1 calcification extending over ≥2 slices[a]	Calcification involving a large segment of a coronary artery branch

[a]Values are based on standard chest CT protocols with 5-mm section thickness.

Abbreviations: CT, computed tomography; LAD, left anterior descending coronary artery; LCX, left circumflex coronary artery; LM, left main coronary artery; RCA, right coronary artery.

Supplementary Table S2. Univariable Cox Regression Analysis.

Variable	HR	95% CI	*p* value
Age	1.04	1.00–1.07	0.027
Postmenopausal status	2.22	1.19–4.15	0.012
History of ACEI/ARB use	2.90	1.14–7.39	0.026
History of statin use	3.93	1.21–12.75	0.023
Anti-HER2 therapy	2.87	1.57–5.27	0.001
CA 15-3	1.00	1.00–1.01	0.023
HDL-C	0.19	0.07–0.55	0.002
ApoA-I	0.22	0.07–0.89	0.033
Baseline hs-cTnT	1.34	1.19–1.51	<0.001
Follicle-stimulating hormone	1.01	1.00–1.02	0.015
CAC score	1.52	1.32–1.76	<0.001
TAC score	1.64	1.40–1.91	<0.001

Abbreviations:HR, hazard ratio; CI, confidence interval; ACEI/ARB, angiotensin-converting enzyme inhibitor/angiotensin receptor blocker; HER2, human epidermal growth factor receptor 2; CA 15-3, cancer antigen 15-3; HDL-C, high-density lipoprotein cholesterol; ApoA-I, apolipoprotein A-I; hs-cTnT, high-sensitivity cardiac troponin T; CAC, coronary artery calcification; TAC, thoracic aortic calcification.

Supplementary Table S3. Incremental predictive value of TAC for 6-month myocardial injury.

Cohort	Metric	Model 1	Model 2	Increment
Training	C-index	0.784	0.804	0.020
Validation	C-index	0.874	0.882	0.008
Training	AUC	0.799	0.810	0.011
Validation	AUC	0.870	0.901	0.031

Model 1 included anti-HER2 therapy, HDL-C, and baseline hs-cTnT. Model 2 additionally included TAC score. Validation performance was assessed using coefficients derived from the training cohort without model refitting. The likelihood-ratio test comparing Model 1 and Model 2 in the training cohort yielded $\chi^2 = 6.62$ (df = 1, $p = 0.010$).

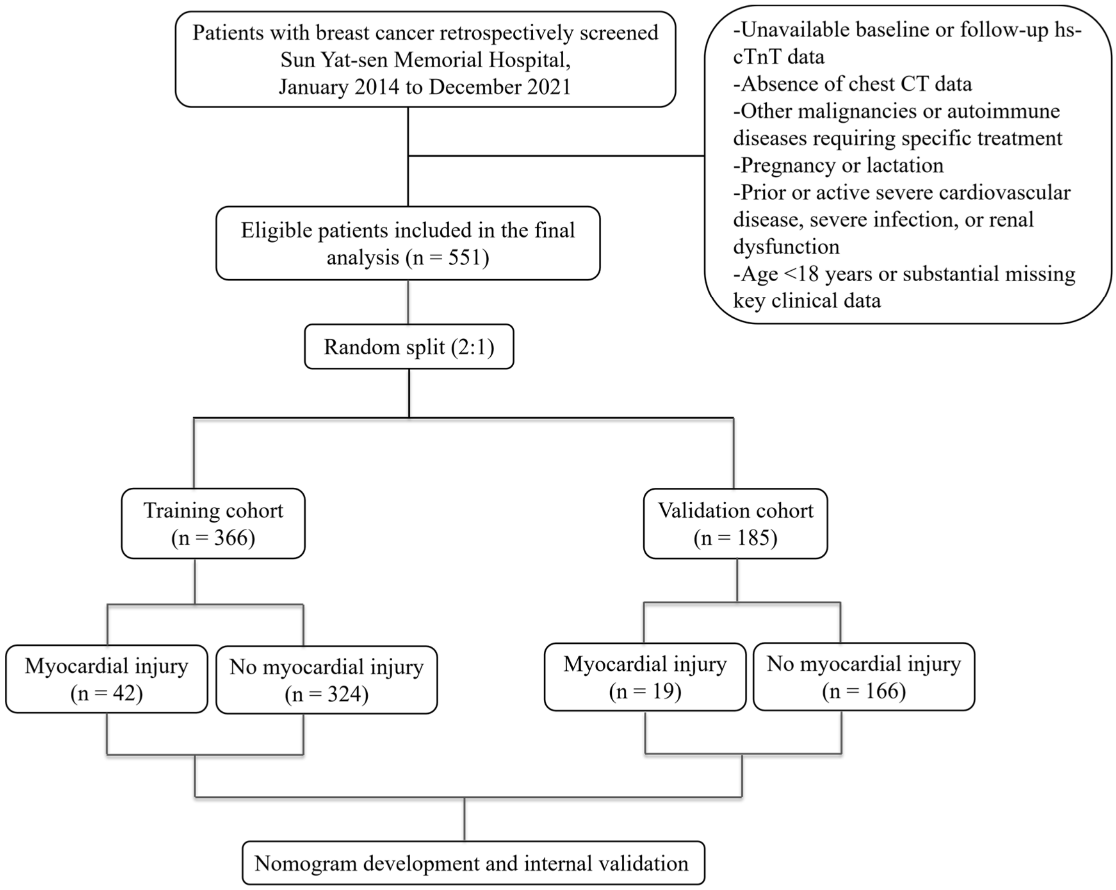

Supplementary Figure S1. Flowchart of patient selection and study design.

Myocardial injury was defined as hs-cTnT >14 ng/L within 6 months after treatment. CT, computed tomography; hs-cTnT, high-sensitivity cardiac troponin T.
